# Non-Syndromic Spinal Schwannomas: A Novel Classification

**DOI:** 10.3389/fneur.2017.00318

**Published:** 2017-07-17

**Authors:** Ibrahim Sun, M. Necmettin Pamir

**Affiliations:** ^1^Department of Neurosurgery, Acıbadem University School of Medicine, Istanbul, Turkey

**Keywords:** spinal schwannoma, resection surgery, tumor classification, postoperative recovery, spinal tumor

## Abstract

Schwannomas are the most frequent primary tumors of the spine with an incidence of 0.3–0.5/100,000 person per year. Current treatment for non-syndromic spinal schwannomas is total resection of the tumor with preservation of neurovascular structures. This study aims to report neurologic and radiologic outcome following treatment of non-syndromic spinal schwannomas along with a novel tumor classification used in our clinic. A retrospective case series was carried out with a patient sample of 82 male and female patients with non-syndromic spinal schwannomas. All patient data were retrospectively collected from the hospital records. As a routine procedure, after admittance and primary evaluation, patients’ tumors were classified using CT or MRI in accordance with our proposed classification method, which employs a dual designation method with tree groups (A, B, and C) for tumor volume and four types (I, II, III, and IV) for tumor localization. Subsequent resection surgery was followed by neurological assessments and follow up at 45th, 180th, and 360th postoperative day. Along with Karnofsky performance status scale, pain, sensory deficits, and motor weakness were scored to assess neurologic recovery. Our finding indicates that patients with different tumor types significantly differ in their neurological scores and show consistent but differential neurological recovery at early and late time points postsurgery. Complications during and postsurgery were minimal, occurring only in two patients. Our findings further reinforce the established safety of total resection operations and indicate that our proposed classification is a simple, effective tool that has proven helpful in preoperative planning and avoiding unnecessary surgical approaches.

## Introduction

Schwannoma is the most common nerve sheat tumor. The incidence of spinal schwannoma is 0.3–0.5/100,000 individuals annually (1). Its prevalence is similar in males and females, and it is usually diagnosed during the fourth and fifth decades of life (2). Schwannomas commonly occur in the lumbar and cervical regions and originate from Schwann cell progenitors (3). Schwannomas are benign tumors that are typically round, well demarcated, and encapsulated. Multiple schwannomas in a patient are referred to as schwannomatosis. Schwannomatosis is usually indicative of an underlying tumor predisposition syndrome, such as neurofibromatosis (4). Among schwannoma patients, 3–4% has multiple tumors (schwannomatosis) (5). Earlier findings suggest that schwannomatosis is a disease that is distinct from non-syndromic schwannomas, both genetically and clinically (6) and, therefore, should be treated accordingly.

Patients with non-syndromic spinal schwannoma usually present to hospital with local pain and neurological deficit that exacerbate over time. Currently, the standard treatment is gross total resection (GTR) of the tumor with as much preservation of neurovascular structures as possible (7). In 1888, Victor Horsley successfully removed an intraspinal tumor (located at the sixth and seventh thoracic vertebra) for the first time (8). In the midst of such advances, preoperative planning remains crucial for successful treatment and relies—to a great extent—on proper tumor classification. The literature includes multiple classification systems for spinal schwannomas, each of which is associated with both positive and negative ramifications for preoperative planning (9–12). Consequently, there is a lack of consensus concerning the optimal system of classification for schwannomas.

The present study aimed to report on the utility of the novel schwannoma classification system used by our neurosurgery department, based on a 27-year review of non-syndromic spinal schwannoma cases that were surgically treated. A review of the literature is also included to provide a detailed overview and comparison of the success rates for other current treatment methods and classification systems.

## Materials and Methods

### Patients

The study included 82 patients that were surgically treated between 1987 and 2015. All patient data were retrospectively obtained from the hospital records and were classified with respect to clinical presentation, radiologic features, and surgical outcomes tumor classification was performed based on CT findings. CT and myelography were used to diagnose schwannomas until 1990; thereafter, diagnosis was performed using MRI. MRI images were not available for three patients, and their tumors were classified based on CT findings.

### Classification

Tumors were classified according to our novel classification system, which is based on consideration of tumor volume and localization relative to the dura and spinal canal. For approximate calculation of tumor volume, spinal schwannomas were considered ellipsoid bodies, and tumor volume was calculated using the following formula:

$$\begin{array}{c} \text{tumor volume}=4/3\;\pi \times (\text{craniocaudal length}/2)\times {\left(\text{transverse diameter}/2\right)}^{2}. \end{array}$$

**Figure 1:**
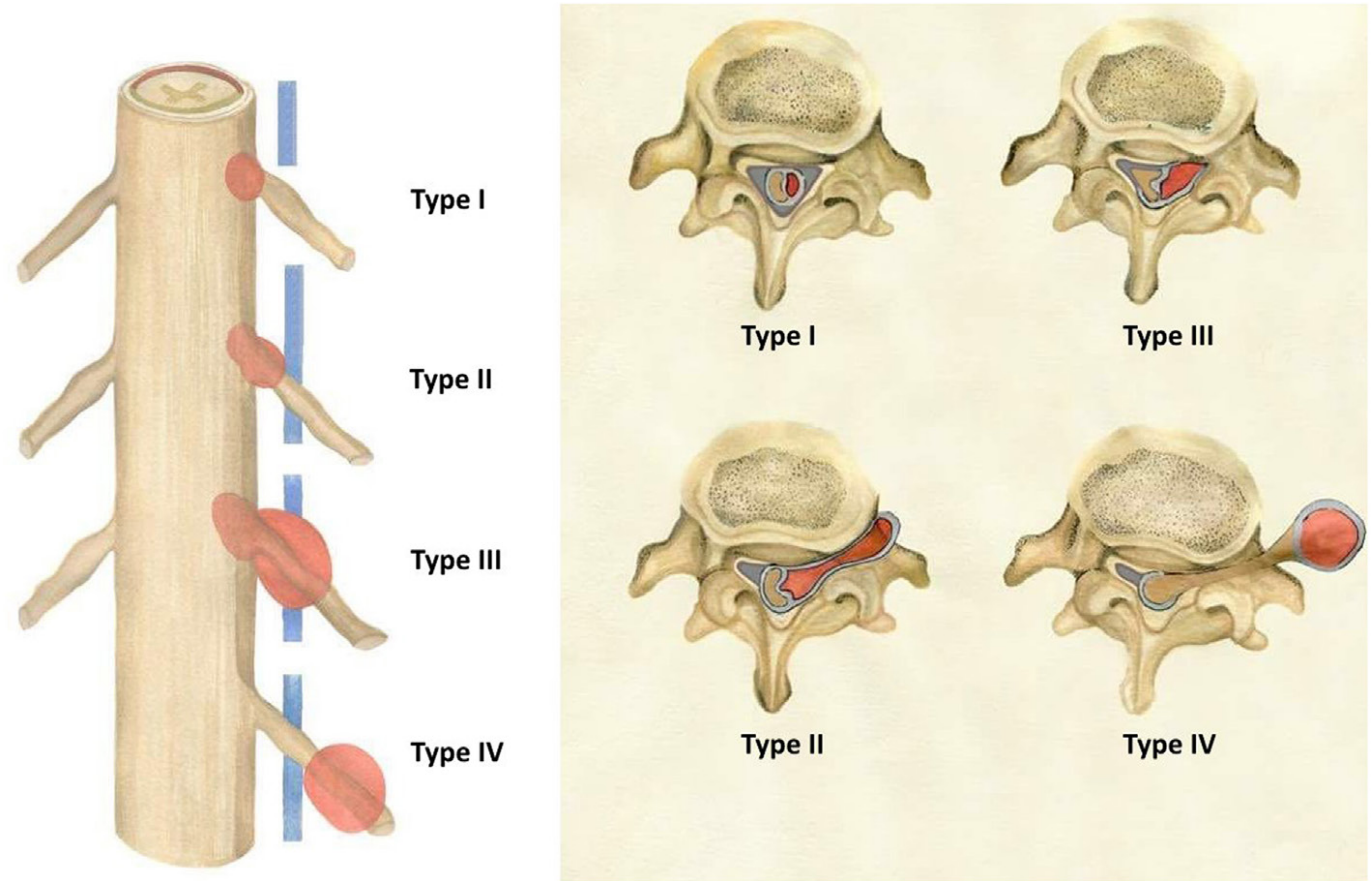
Schwannomas types (according to the present study’s novel classification system) as seen in the coronal and transverse planes.

Tumors were then assigned to 1–3 volume groups (group A, B, and C) and designated as 1 of 4 types (type I, II, III, and IV) according to localization (i.e., group B type II tumor). Tumor volume <2 cm^3^ was considered group A, 2–4 cm^3^ group B, and >4 cm^3^ group C. Tumor typing was as follows: localized exclusively intradurally: type I; intradural localization with extradural extension to the nerve root foramina, but restricted to the spinal canal: type II; intradural dumbbell-shaped tumor in the spinal canal extending to the extraforaminal region: type III; and localized completely outside the root foramina: type IV (Figure 1).

### Surgery

All tumors were removed *via* a posterior approach. Laminectomy was performed over the appropriate number of levels; where necessary this was expanded to include the medial facet. Ultrasonic aspiration was used to facilitate tumor debulking. The aim of the surgery was to remove the tumor while preserving the parent nerve. For type III tumors, intracapsular resection was performed *via* CUSA, followed by dissection performed from the intradural to extradural side. Bone removal is not necessary in cases of type IV tumors, and in such cases direct tumor exposure was performed. The aim of all surgeries was GTR and preservation of the parent nerve. Tumor resection was performed microsurgically, and an ultrasonic aspirator was used for enucleation. Among the patients included in the study, intraoperative monitorization with somatosensorial-evoked potentials was used in 57 (70%) that underwent surgery after 2000.

### Follow-up

Gross total resection was confirmed in 79 patients based on MRI performed 24 h post surgery. Follow-up was performed using CT in three patients prior to 1990, as MRI was not available at that time. Follow-up examinations were performed 45 days, 3 months, and 1 year post surgery, and then yearly for the next 4 years (total: 5 years), but follow-up results for the first postsurgery year only are included in the study. For statistical analysis, each patient’s clinical course was scored *via* the Klekamp and Samii (13) scoring system, which was designed for all types of spinal schwannomas considered by the novel classification system used in the present study. Pain, sensory deficit, motor weakness, and Karnofsky scores were recorded in each patient before surgery, 24 h post surgery, and 45 days, 3 months, and 1 year post surgery (Table 2).

### Statistical Analysis

Patient results were summarized with mean, SD; whereas categorical variables were summarized with count and percentage. Preoperative, postoperative 45th day, 3rd month, and 1st year results were compared among different types (according to our classification) of spinal schwannomas. One-way between-subject ANOVA was run since the design aimed to analyze the mean differences between the types of tumors according to our classificatory system, *k* = 4.

### Ethical Consideration

The study protocol was approved by the Acıbadem University Ethics Committee, Istanbul, Turkey. All patients provided written informed consent for use of their data in this study.

## Results

**Figure 2:**
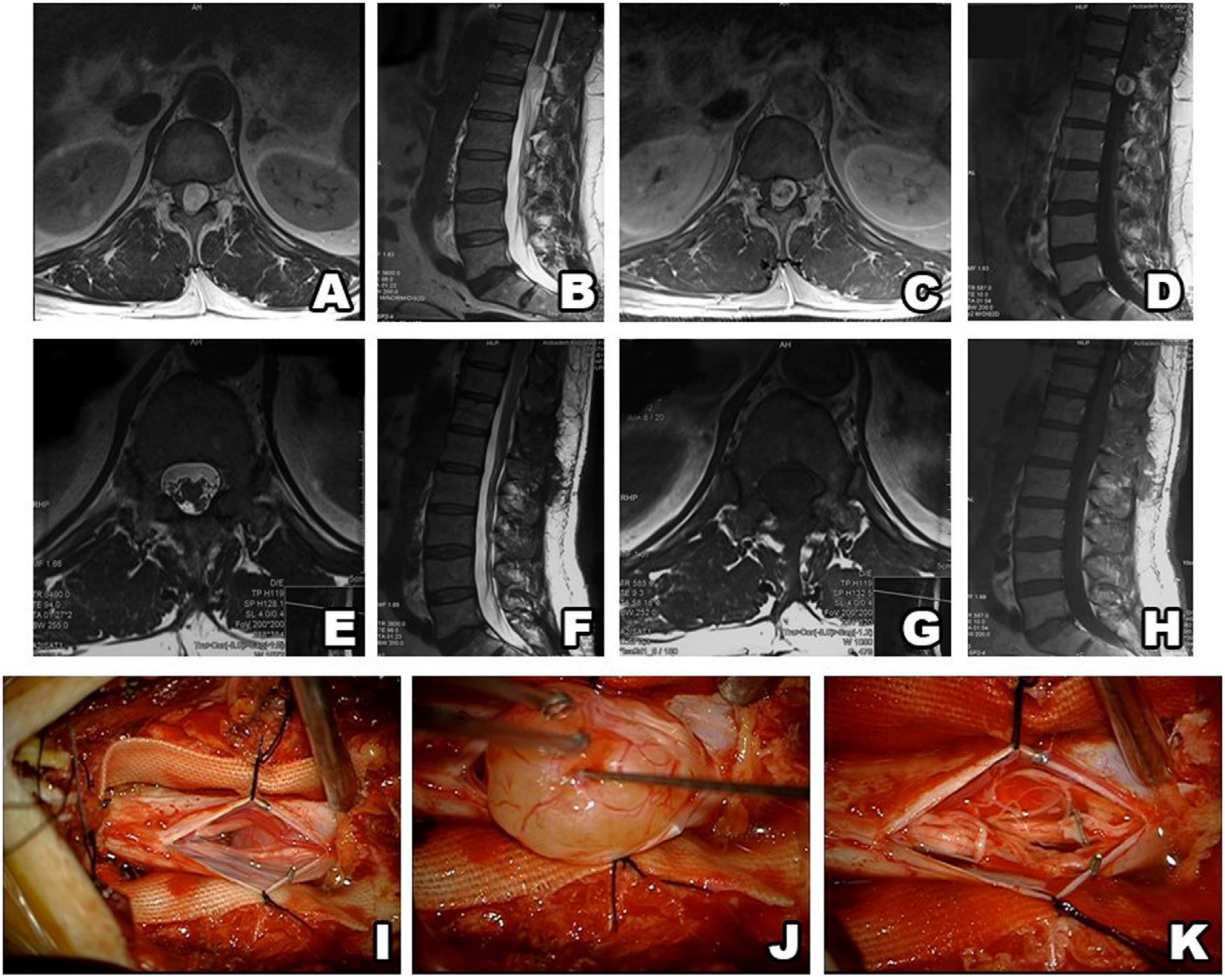
Type I schwannoma (intradural localization). MRI shows a heterogeneous, contrast-enhanced intradural tumor **(A–D)**. Postsurgery MRI confirms gross total resection, without hematoma or recurrence **(E–H)**. Intraoperative photos before and after tumor resection **(I–K)**.

**Figure 3:**
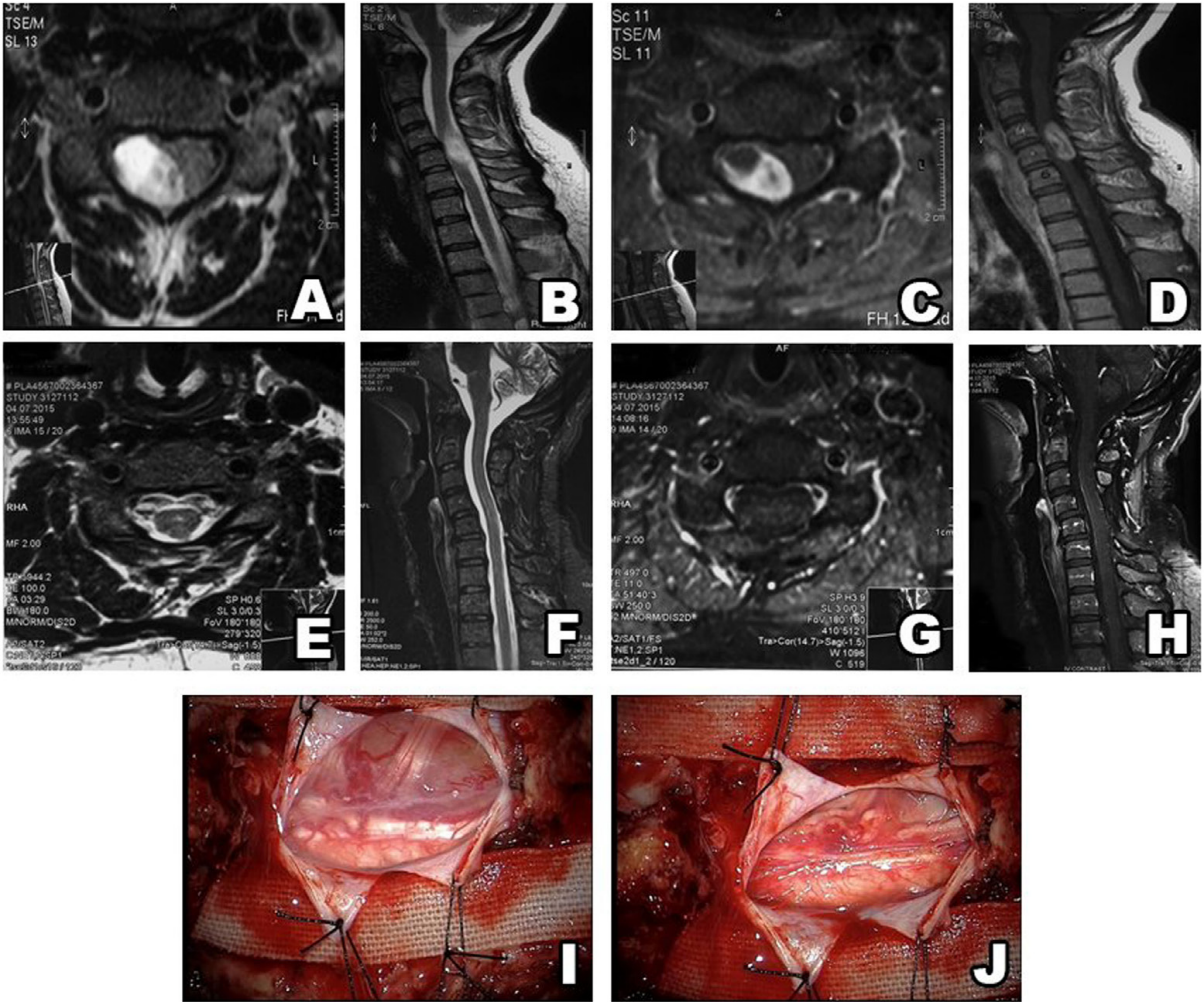
Type II schwannoma (intradural localization). MRI shows a heterogeneous, contrast-enhanced intradural tumor **(A–D)**. Postsurgery MRI confirms gross total resection, without hematoma or recurrence **(E–H)**. A schwannoma inside the dura **(I)**. After resection, there is no residue remaining in surgical field **(J)**.

**Figure 4:**
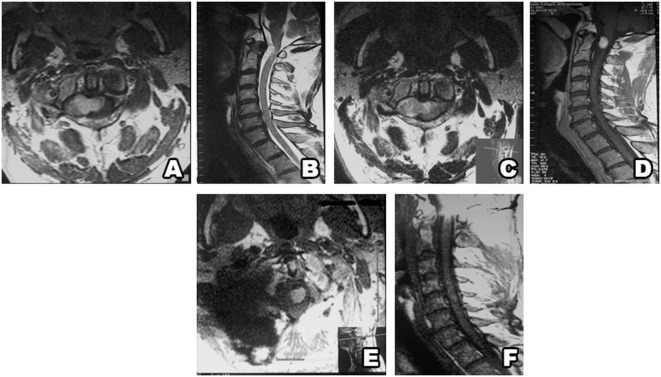
Type II schwannoma (intradural dumbbell-shaped tumor with extension to the extraforaminal region) **(A,B)**, and with contrast enhancement **(C,D)**. Postsurgery MRI confirms gross total resection, without hematoma or recurrence **(E,F)**.

**Figure 5:**
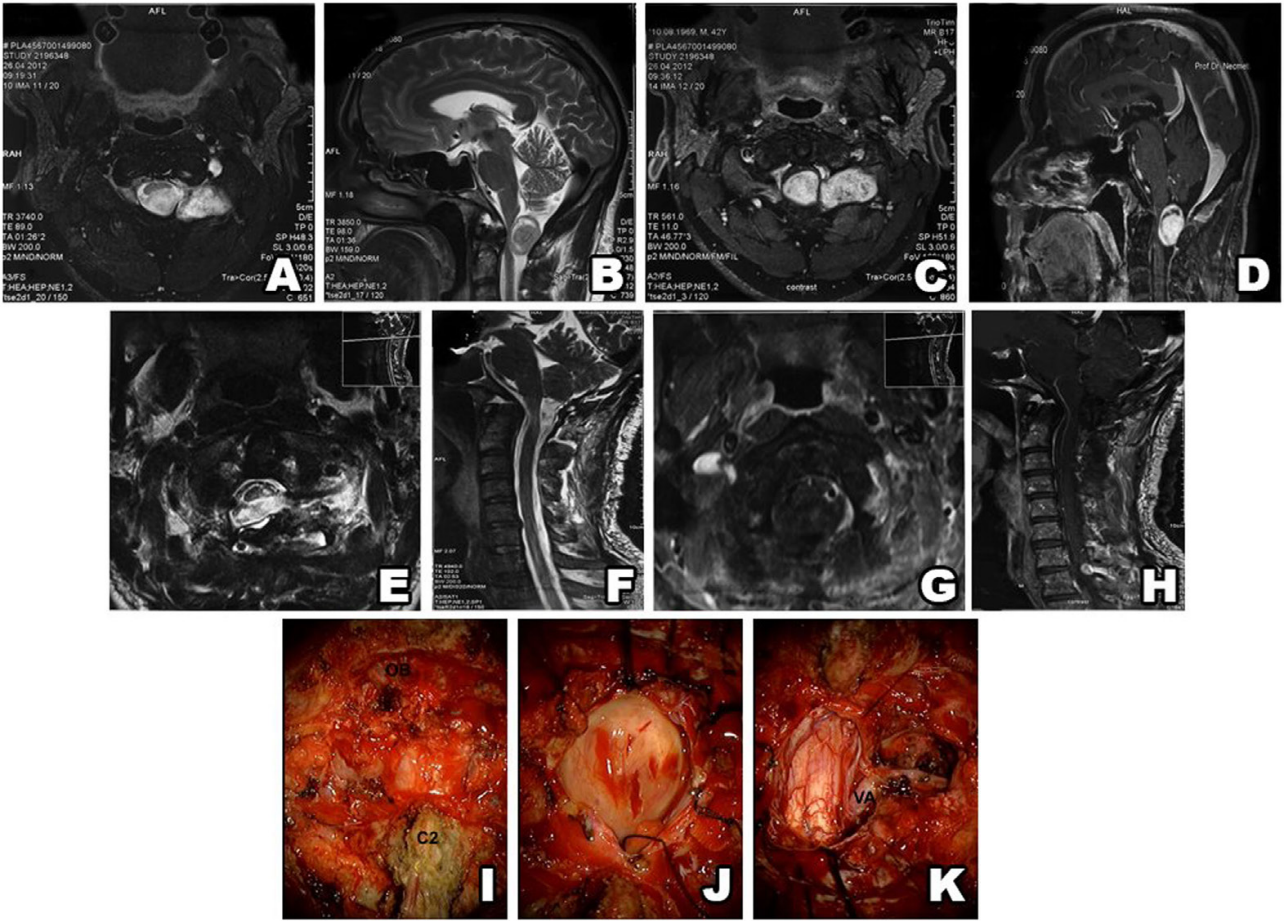
Type III schwannoma (intradural dumbbell-shaped tumor within the spinal canal and extending to the extraforaminal region). Preoperative MRI shows a tumor with extraforaminal extension **(A–D)**. Postsurgery MRI confirms gross total resection, without hematoma or recurrence **(E–H)**. Intraoperative view of a schwannoma extending beyond the spinal canal **(I)** (OB, occipital bone; C2, second cervical vertebra). Intradural part of a schwannoma **(J)**. Postoperative view of the surgical field shows no tumor residue **(K)** (VA, vertebral artery).

**Figure 6:**
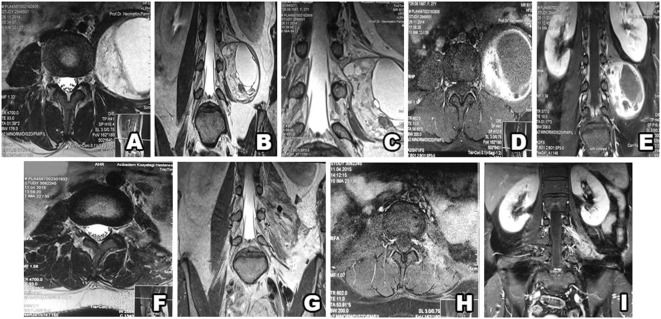
Type IV schwannoma (tumor residing completely outside the root foramina). Preoperative MRI shows a giant cystic schwannoma **(A–E)**, with heterogeneous color enhancement **(D,E)**. Postsurgery MRI confirms gross total resection, without hematoma or recurrence **(F–I)**.

**Figure 7:**
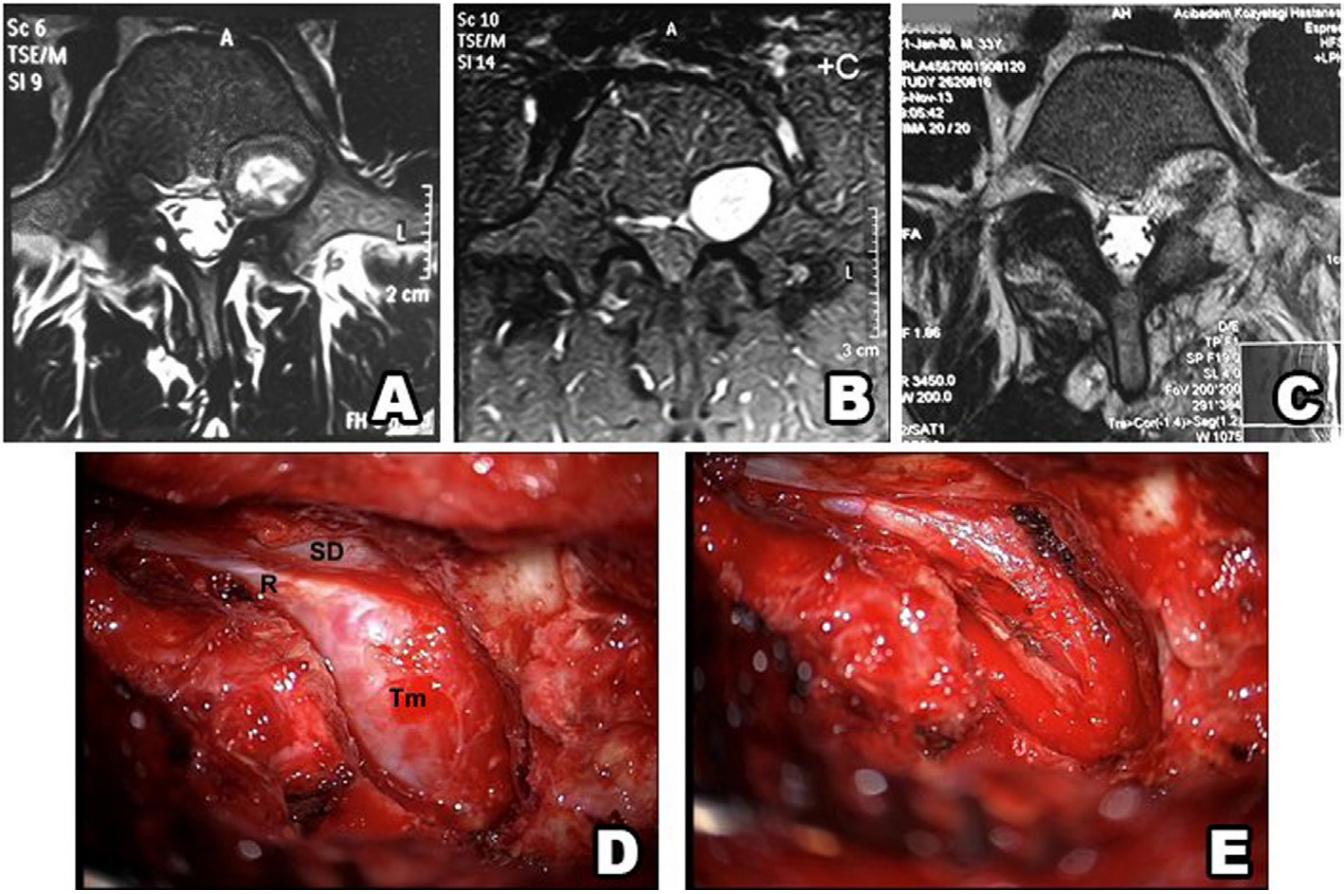
Type IV schwannoma (tumor residing completely outside the root foramina). Preoperative MRI shows a giant cystic spinal schwannoma **(A–C)**. An extraforaminal tumor in the surgical field **(D)** (SD, spinal dura; R, root; Tm, tumor). Postoperative view of the surgical field shows no tumor residue **(E)**.

The study included 82 patients with non-syndromic spinal schwannoma. Cystic components were noted in 10 of the patients. The surgical procedure was the same in patients with and without tumors with cystic components. The patient population consisted of 39 (47.5%) females and 43 (52.5%) males, with a mean age of 45.4 years (range: 18–77 years). In all, almost half of the patients’ spinal schwannomas were group B (volume of 2–4 cm^3^). Based on MRI, schwannomas are well-circumscribed, appear isointense to hypointense in T1-weighted images and primarily hyperintense in T2-weighted images, and exhibit various patterns of contrast enhancement. GTR was achieved in 81 patients versus incomplete resection in 1 patient in which vertebral artery injury occurred during resection of the anterior portion of the tumor. Schwannoma localization was cervical in 34 (43%) patients, thoracic in 17 (21%), and lumbar in 31 (36%). According to our novel classification system, based on tumor volume the tumors were categorized as group A in 25 (30%) patients, versus group B in 36 (44%) and group C in 21 (26%) (Table 1). The most common schwannoma type was type I [*n* = 42 (51%)] (Figures 2 and 3), followed by type II [*n* = 24 (29%)] (Figure 4), type III [*n* = 10 (12%)] (Figure 5), and type IV [*n* = 6 (8%)] (Figures 6 and 7). Symptoms at presentation were pain in 65 patients (79%), paresthesia and/or numbness in 43 (52%), and motor weakness in 21 (26%) (Table 2). In all, two patients had postoperative lumbar drainage, which did not cause infection.

**Table 1:** Frequencies for tumor size and location of patients according to our proposed classification.

		Cervical, *n* (%)	Thoracic, *n* (%)	Lumbar, *n* (%)	Total
Type I	A	3 (3.6%)	4 (4.9%)	12 (14.9%)	19 (23.1%)
	B	2 (2.4%)	8 (9.9%)	6 (7.3%)	16 (19.5%)
	C	3 (3.6%)	4 (4.9%)	–	7 (8.5%)

Type II	A	4 (4.9%)	–	2 (2.4%)	6 (7.3%)
	B	6 (7.3%)	1 (1.2%)	3 (3.6%)	10 (12.2%)
	C	6 (7.3%)	–	2 (2.4%)	8 (9.9%)

Type III	A	–	–	–	–
	B	4 (4.9%)	–	–	4 (4.9%)
	C	5 (6.1%)	–	1 (1.2%)	6 (7.3%)

Type IV	A	–	–	–	–
	B	1 (1.2%)	–	5 (6.1%)	6 (7.3%)
	C	–	–	–	–

Total		34 (41.4%)	17 (20.9%)	31 (37.3%)	82 (100%)

**Table 2:** Patients’ neurological scores of with different tumor types at preoperative, postoperative, and follow-up time points.

	Type I (*n* = 42)	Type II (*n* = 24)	Type III (*n* = 10)	Type IV (*n* = 6)	*P*
**Pain**					
Preoperative	2.8 ± 0.2	3.4 ± 0.4	3.6 ± 0.6	4.0 ± 0.7	<0.001
24 h	3.0 ± 0.2	4.1 ± 0.3	4.8 ± 0.3	4.2 ± 0.8	<0.001
45 days	3.5 ± 0.2	4.2 ± 0.2	4.1 ± 0.4	4.5 ± 0.6	<0.001
3 months	3.7 ± 0.2	4.2 ± 0.3	4.2 ± 0.5	4.7 ± 0.5	<0.001
1 year	3.6 ± 0.2	4.4 ± 0.2	4.5 ± 0.4	4.7 ± 0.5	<0.001
**Sensory deficit**					
Preoperative	3.7 ± 0.2	2.4 ± 0.3	2.8 ± 0.6	3.3 ± 0.9	<0.001
24 h	3.9 ± 0.2	3.5 ± 0.3	3.5 ± 0.5	3.5 ± 0.9	0.121
45 days	4.0 ± 0.2	3.7 ± 0.2	3.6 ± 0.5	3.8 ± 0.8	0.277
3 months	4.1 ± 0.2	4.2 ± 0.2	4.0 ± 0.3	4.0 ± 0.7	0.752
1 year	4.2 ± 0.2	4.2 ± 0.2	4.2 ± 0.5	4.0 ± 0.7	0.922
**Motor weakness**					
Preoperative	3.1 ± 0.2	4.0 ± 0.3	3.3 ± 0.6	4.2 ± 0.8	<0.001
24 h	4.2 ± 0.2	3.1 ± 0.3	3.4 ± 0.6	4.2 ± 0.8	<0.001
45 days	4.3 ± 0.2	4.1 ± 0.2	3.8 ± 0.6	4.7 ± 0.5	0.043
3 months	4.2 ± 0.2	4.2 ± 0.2	3.9 ± 0.5	4.7 ± 0.5	0.141
1 year	4.7 ± 0.1	4.4 ± 0.2	3.5 ± 0.5	4.7 ± 0.5	<0.001
**Karnofsky score**					
Preoperative	74.3 ± 2.9	72.1 ± 3.1	73.0 ± 3.5	70.0 ± 6.6	0.538
24 h	77.9 ± 2.3	76.7 ± 2.4	78.0 ± 4.5	76.7 ± 5.4	0.890
45 days	79.8 ± 2.1	80.0 ± 2.8	81.0 ± 6.3	83.3 ± 5.4	0.671
3 months	88.1 ± 2.2	84.6 ± 3.1	84.0 ± 5.0	86.7 ± 5.4	0.163
1 year	89.8 ± 2.2	87.9 ± 2.8	82.0 ± 2.8	88.3 ± 7.9	0.017

*95% confidence intervals and P value for non-significant results for P = 0.05 are presented. One-way between-subjects ANOVA is used*.

Preoperative pain, sensory deficit, and motor weakness scores differed significantly according to schwannoma type (*P* < 0.001 in all tumor groups). The Karnofsky score were improved in all the patients at all postsurgery time points (*P* > 0.05), but only significantly at 1 year post surgery (*P* < 0.017) (Table 2). Only one patient developed postoperative wound infection and was treated with antibiotics. Another two patients had a CSF fistula that was treated with lumbar drainage, after which time they were removed without any complication. In all, two patients showed signs of clinical deterioration after surgery, but both recovered within 2 weeks. At 1 year post surgery all the patients were alive.

## Discussion

Spinal schwannomas account for approximately 25% of all spinal tumors and are the most common nerve sheath tumors (3, 11). Mitotic figures are rarely observed in schwannomas (14). Similar to earlier reports, in the present study cervical and lumbar schwannoma localization was more common than thoracic (1). MRI facilitates easy differentiation of extradural, intradural-extramedullary, and intramedullary schwannomas (15) and is routinely used to examine spinal schwannomas by our department. Preoperative management and postoperative monitorization for schwannoma recurrence are performed using MRI in our department.

Depending on localization, patients with spinal schwannomas primarily present with radicular pain and radiculopathy. Numbness, paresthesia, and motor weakness may also be present, but in the present study the initial symptom was pain in 65 (79%) of the patients. In the present study, pain, sensory deficit, motor weakness, and Karnofsky scores did not improve in those with type I and type IV tumors. In patients with type II and type III tumors, improvement was observed after surgery, but it was not significant. Spinal schwannomas primarily originate from the sensorial division of the nerves, and these data support why the sensory deficit score did not improve significantly for all types of tumors. As compared to presurgery, the Karnofsky score 24 h post surgery did not improve significantly for any type of schwannoma; however, at 1 year post surgery, it improved significantly for every type.

Whether or not to perform GTR and sacrifice the functionally important root remains a critical decision for surgeons. Kim et al. (12) reported 31 cases that involved the functionally important root (C5-T1 or L3-S1) in which GTR was achieved by sacrificing the root. They reported a neurologic deficit rate of 23%, but that the observed deficit was not functionally debilitating in any of the cases. In the present study, none of the patients had additional nerve deficit after scarifying the nerves affected by schwannoma.

Sarcomatous degeneration has been reported in patients with neurofibromatosis (15). Molecular and genetic studies show that schwannomatosis is a distinct genetic and clinic syndrome (16); therefore, schwannomatosis patients with neurofibromatosis were excluded from the present study. Management of multiple schwannomas is often considerably more complex than that for solitary tumors (4). Retroperitoneally localized schwannomas are rare, comprising approximately 3% of all schwannomas (8). Similarly, the present study included just one patient with a retroperitoneal schwannoma. The correlation between tumor volume and MRI findings in the present study shows that type IV tumors were larger than the other types of tumors and that they did not have margins surrounded by bones, as did intraspinal canal tumors. Intradural tumors become symptomatic sooner than other schwannoma types because they compress the spinal cord. In addition, intradural schwannomas are smaller than the other schwannoma groups in the diagnosis because of this location-dependent pain is a cause to see the physician.

A search of the schwannoma literature was performed for comparison with the present findings. In all, 25 relevant studies published between 1992 and 2016 were found. According to the literature, 2,412 spinal schwannomas cases (including those in the present study) have been reported (Tables 3 and 4). The median of number cases per study was 44, 1,247 (51.69%) cases were male and 1,165 (48.31%) were female, and mean age was 45.6 years. Tumor localization among the 2,142 cases was as follows: cervical: 33.92%; thoracic: 28.4%; lumbosacral: 37.68%. Data on the extent of surgical resection were available for 2,405 of the cases, and the GTR rate was 93%. Postsurgery functional and neurological scores were reported for 2,138 of the patients; in 79.84% they increased, in 14.4% they remained the same, and in 5.76% they were lower, as compared to presurgery.

**Table 3:** General characteristics of the 25 non-syndromic schwannoma studies published between 1992 and 2015.

Study	Patients (*n*)	Male to female ratio	Mean age at diagnosis (years)	Cervical (*n*)	Thoracic (*n*)	Lumbosacral (*n*)	GRT rate (%)	Outcome worse (*n*)	Outcome same (*n*)	Outcome better (*n*)
Friedman et al. (17)	7	3/4	46.2	1	2	4	N/A	N/A	N/A	N/A
Seppala et al. (1)	187	83/104	48	43	54	90	90.1	10	61	116
Asahara et al. (18)	42	17/25	45.1	12	18	12	97	N/A	N/A	N/A
McCormick (19)	12	6/6	48	0	6	6	83.3	0	0	12
Domínguez et al. (20)	6	1/5	45	0	0	6	83.3	0	1	5
Iwasaki et al. (11)	4	1/3	41	4	0	0	100	0	0	4
Subaciute (16)	76	25/51	47.9	19	46	11	98	0	1	75
Asazuma et al. (21)	42	24/18	42	48	0	0	85.7	1	11	36
Conti et al. (22)	152	59/93	44.3	33	49	70	95	3	17	132
Safavi-Abbasi et al. (3)	128	76/52	47.7	37	34	57	97	16	0	112
Jeon et al. (23)	38	22/16	50.2	4	11	25	95	0	3	35
Jiang et al. (24)	44	29/15	42.3	44	0	0	100	8	0	36
Raysi Dehcordi et al. (25)	16	5/11	51	16	0	0	100	0	0	16
Fernandes et al. (10)	30	15/15	40	7	10	13	93.4	28	1	1
Chowdhury et al. (9)	15	8/7	35.8	15	0	0	100	3	1	11
Altas et al. (26)	35	15/20	47.2	7	10	18	96	N/A	N/A	N/A
Yamane et al. (27)	30	18/12	48	30	0	0	53	N/A	N/A	N/A
Deng et al. (28)	52	25/27	47.5	2	23	27	100	0	1	51
Halvorsen et al. (29)	131	77/54	47	39	23	69	86	16	5	110
Turel et al. (30)	164	109/55	42.6	46	77	44	92	11	39	117
Lee et al. (31)	49	25/24	45	15	12	22	96	5	0	44
Zamorano et al. (32)	169	88/81	46.6	17	48	104	100	N/A	N/A	N/A
Pompili et al. (33)	70	34/36	52.2	6	27	37	98.6	0	3	67
Li et al. (34)	831	443/388	44.8	343	221	267	93.9	21	144	666
Pamir*	82	39/43	45.4	34	17	31	98.7	1	20	61

Total	2,412	1,247/1,165	45.6	822	688	913	93	123	308	1,707

*Current research to compare others.

**Table 4:** The general characteristics of the 2,412 spinal schwannoma cases published between 1992 and 2015 (including the present study).

Schwannoma cases published between 1992 and 2016 (*n* = 2,412)		

Gender	*n*	%
Male	1,247	51.69
Female	1,165	48.31

**Mean age at diagnosis (years)**		45.6

**Tumor location**	**Tumors (*n*)**	**%**

Cervical	822	33.92
Thoracic	688	28.39
Lumbosacral	913	37.68

**Gross complete resection**	2,310	93.00

**Outcome**	**Patients (*n*)**	**%**

Better	1,707	79.84
Same	308	14.40
Worse	123	5.75

The methods used to assess neurological outcome post surgery vary from study to study; as such, comparison is difficult. Nevertheless, the percentage of patients with functional and neurological scores that were lower post surgery might be a reliable measure for evaluating the surgical success rate, as lower functional and neurological scores post surgery are generally due to complications. Studies from 1992 to 2015 reported lower functional and neurological scores post surgery in approximately 5.75% of patients versus a significantly lower 1.2% in the present study (which also correlates with the number of patients with complications). As the GTR rate according to meta-analysis was 93%, reducing the incidence of postoperative side effects is an area to achieve. The novel schwannoma classification system described herein decreased the number of patients with lower functional and neurological scores post surgery by 25%. Establishing and standardizing best practices in the surgical theater is a never-ending challenge for all surgery centers. A plethora of factors, ranging from surgeon skill to surgical theater conditions, affect surgical outcome. In addition, presurgery planning is crucial for reducing the incidence of surgical complications. Based on the present findings, we think that the present study’s low complication rate is associated with our standardized preoperative planning phase, which is based on our novel schwannoma classification system.

The literature includes numerous schwannoma classification systems. Jinnai and Koyama (2) classified schwannomas into five groups based on the relationship between the tumor and the dura mater and/or intervertebral foramen. This classification system is useful, as it takes into consideration tumor localization relative to the dura, but it does not take into account volume, which is important for preoperative surgical planning. Asazuma et al. (21) devised a schwannoma classification system for cervical dumbbell-shaped tumors that consisted of nine categories. An important drawback of their classification system is that it cannot be used for thoracic or lumbar schwannomas, which are as common as cervical schwannomas. Sridhar et al.’s (35) classification system is arguably the most similar of the previously reported systems to the novel classification system described herein; however, we think classification of seven distinct types of schwannomas using Sridhar et al.’s system is not practical because the characteristics of seven tumors types are difficult to remember. Another drawback of their system is that tumor volume is only considered for dumbbell-shaped tumors, and craniocaudal dimension is not a consideration, which limit the diagnostic value and consistency of the classification system. Based on the present findings, we think that all schwannomas should be classified according to localization and volume, so as to achieve the desired benefit of classification—ease and reliability of preoperative decision making and preparation. In addition, our novel spinal schwannoma classification system makes tumor localization easier to understand, as compared to other systems, and is suitable for all schwannoma types.

The present findings help confirm that GTR of non-syndromic spinal schwannomas is safe and effective. Spinal schwannomas should be evaluated *via* MRI both pre- and postsurgically. Standardized tumor classification prior to any intervention is highly desirable and helpful for surgical planning. Tumor types should be carefully evaluated for optimal surgical planning. The novel spinal schwannoma classification system described herein is a simple and effective tool that the present findings show extremely helpful for avoiding unnecessary surgical approaches and complications. Due to the system’s simplicity of having only three tumor groups and its reliability—indicated by the associated low postoperative side effect rate, use of this novel classification system should be considered by any surgical department that seeks a standardized schwannoma surgery protocol.

## Ethics Statement

This study was carried out in accordance with the recommendations of Acıbadem University Ethical Committee of Medical Research with written informed consent from all subjects. All subjects gave written informed consent in accordance with the Declaration of Helsinki. The protocol was approved by the “Acıbadem University Ethical Committee of Medical Research.”

## Author Contributions

All authors contributed equally to this work.

## Conflict of Interest Statement

The authors declare that the research was conducted in the absence of any commercial or financial relationships that could be construed as a potential conflict of interest.
